# Seasonal variation of 25-Hydroxyvitamin D and indices of carbohydrate and lipid metabolism in postmenopausal women

**DOI:** 10.7717/peerj.11341

**Published:** 2021-05-13

**Authors:** Anna Huta-Osiecka, Krystian Wochna, Zbigniew Kasprzak, Alicja Nowak

**Affiliations:** 1 Department of Hygiene, Poznań University of Physical Education, Poznań, Poland; 2 Laboratory of Swimming and Water Lifesaving, Poznań University of Physical Education, Poznań, Poland

**Keywords:** Vitamin D, Fat mass, Insulin sensitivity, Lipid profile, Postmenopausal women, Seasonal variation

## Abstract

**Background:**

Some studies indicate vitamin D’s significant contribution to metabolic processess. Therefore, the purpose of this study was to evaluate the level of carbohydrate and lipid metabolism indices in relation to seasonal changes in 25-hydroxyvitamin D (25(OH)D) concentration in postmenopausal women.

**Methods:**

Sixteen postmenopausal women meeting health criteria and not using vitamin D supplementation were included in the study. Seasonal variation of somatic features and the serum concentration of 25(OH)D, glucose, insulin, parathormon, sclerostin and lipid profile were determined on two terms (autumn-winter).

**Results:**

Comparative analysis of the variables between the study terms revealed a marked decrease in the concentration of 25(OH)D (*p* ≤ 0.0001), insulin (*p* < 0.05), insulin resistance index (HOMA-IR), (*p* < 0.05). The significant positive correlations of changes (Δ) between autumn and winter in 25(OH)D with body mass (*p* < 0.05), and fat mass (*p* ≤ 0.01), measured in the first study term, in the group of women with normal body mass index (BMI), and negative correlation with fat mass (*p* < 0.05) in women with a BMI value above the reference values, were found. The relationship analysis showed that in women with normal BMI, the decrease in 25(OH)D concentrations was greater when the body fat percentage was higher, whereas in women with a BMI value above the reference values, the higher the fat percentage, the smaller was the decrease in 25(OH)D concentration.

**Conclusions:**

Seasonal changes in 25(OH)D concentration did not significantly affect the concentration of carbohydrate and lipid metabolism indices. The magnitude of decline in 25(OH)D levels depends on the fat mass. We suppose that environmental or lifestyle-related factors, e.g., nutritional behaviours, may have had more influence on metabolic indices than changes in 25(OH)D.

## Introduction

Vitamin D exhibits pleiotropic effects, supported by the presence of vitamin D receptors (VDRs) in many of the body’s tissues and organs (Christakos et al., 2016). The identification of VDRs in pancreatic cells (Christakos et al., 2016), adipose tissue and the liver (Cimini et al., 2019) allows us to conclude that apart from its known calcitropic functions, it also contributes to energy metabolism and the organism’s energy balance. Some studies indicate vitamin D’s significant contribution to carbohydrate and lipid metabolism (Grimnes et al., 2011; Jungert, Roth & Neuhäuser-Berthold, 2015; Greco, Lenzi & Migliaccio, 2019; Kayaniyil et al., 2010). The population-based study confirmed positive relationships between serum 25(OH)D concentration and insulin sensitivity measured using a 3-h hyperglycemic clamp (Grimnes et al., 2011) or oral glucose tolerance tests (Kayaniyil et al., 2010) and significant relationships with lipid profiles (Grimnes et al., 2011; Jungert, Roth & Neuhäuser-Berthold, 2015).

In humans, vitamin D_3_ is formed in the skin as a result of 7-dehydrocholesterol isomerization, which occurs under the influence of sunlight (UVB) or comes from the diet, especially from fish (Nakamura et al., 2000; Norval, Björn & de Gruijl, 2010). However, it is suggested that only frequent fish consumption may help to supply adequate vitamin D (Nakamura et al., 2000). In epidemiological studies, the status of vitamin D in the human body is assessed by determining the blood level of 25(OH)D, a metabolite resulting from the hydroxylation of vitamin D in the liver. The biologically active form of vitamin D, which activates intracellular nuclear vitamin D receptors (VDRs), is 1,25-dihydroxycholecalciferol (1,25(OH)_2_D_3_), the product of 25(OH)D hydroxylation in the kidneys (Jukic, Hoofnagle & Lutsey, 2018).

The change in UV intensity during the different seasons of the year influences the change in serum 25(OH)D (Papadakis et al., 2015; Andersen et al., 2013). There was, however, no clear explanation of whether seasonal changes in 25(OH)D concentration may be related to changes in carbohydrate and lipid metabolism indices. Seasonal (winter-summer) changes in insulin sensitivity and the level of lipid metabolism indices were observed in earlier studies conducted on elderly people (Berglund et al., 2012; Skutecki et al., 2019). In these studies, the changes in these indices were explained by the variability of the external temperature and changes in diet and body composition.

The aim of the present study is to assess the level of lipid and carbohydrate metabolism indices in the autumn-winter period in relation to seasonal changes in 25(OH)D concentrations in postmenopausal women. Some authors’ studies point to the dependence of 25(OH)D level on the content of adipose tissue (Carrelli et al., 2017; Tosunbayraktar et al., 2005); therefore the influence of this factor was taken into account in the analysis of the research findings.

## Materials & Methods

### Participants and the study protocol

Thirty-eight postmenopausal women declaring good health and not using hormone replacement therapy applied for the study. The survey questionnaire provided information on lifestyle, diseases, drugs and supplements used and the frequency of fish consumption. Based on the data collected at this stage of the study, those who declared the occurrence of diabetes, insulin resistance, liver diseases, using drugs and supplements modifying lipid metabolism, a stay abroad in countries with high levels of sunlight during the two weeks preceding the study and systematic participation in physical activity classes were not qualified for the subsequent stages of the study. The subjects were asked not to take any preparations containing vitamin D and not to introduce changes in their lifestyle (physical activity, diet) during the study period. Participants who failed to abide by the research protocol were excluded. Finally, 16 women aged 62.44 ± 4.76 years were qualified for the study. Before the research was conducted, all subjects were informed of the study’s purpose and methods involved. All participants provided written consented to participating in the study. The research was approved by the Bioethics Committee at the Karol Marcinkowski Medical University in Poznań (code no.901/17).

The research was carried out in the period from autumn to winter (the first term of research—end of September/beginning of October, the second term of research—middle of December (seventy-five days between the two measurements). The study assessed serum concentrations of 25(OH)D and selected indicators of carbohydrate and lipid metabolism.

### Anthropometric and biochemical measurements

During the first and second terms of the study, body composition was assessed and blood was collected for the biochemical analysis. Body composition, body mass and height were measured in a fasting state, using the electrical bioimpedance method (BIA, the Tanita BC 418-MA analyzer) and a certified Radwag device (Radom, Poland) with an accuracy of 0.5 cm. The value of body mass index (BMI) was calculated as body mass (kg)/body height^2^ (m^2^). Based on the BMI scores, groups of women were distinguished using the recommendations of the Committee on Diet and Health, according to which the range of normal BMI values is determined on the basis of the age of subjects (Babiarczyk & Turbiarz, 2012). Two groups of women were formed: group A (*n* = 10) with normal BMI (for age 45–54 y—proper BMI: 22-27 kg/m^2^, for age 55–65 y—BMI:23-28 kg/m^2^, for age >65 y BMI: 24-29 kg/m^2^) and group B (*n* = 6) with BMI above normal range.

The fasting blood samples for biochemical analyses were taken from the antecubital vein, between 7.30 and 9.30 a.m. and then centrifuged to obtain serum. The serum was stored at −70 °C until the biochemical analyses were performed. The serum concentration of 25(OH)D was determined by chemiluminescence immunoassay (CLIA) (LIAISON^®^ 25 OH Vitamin D TOTAL Assay, DiaSorin Inc, USA, sensitivity 4 ng/ml). The concentrations of glucose and lipid metabolism indices (TC, total cholesterol; TG, triglycerides; HDL-C, high-density lipoprotein cholesterol; LDL-C, low-density lipoprotein cholesterol) were determined with the ACCENT 220s analyzer and Cormay tests, Poland (the sensitivity of the tests was 0.41 mg/dl, 1.95 mg/dl, 1.4 mg/dl, 1.1 mg/dl, 3.9 mg/dl, respectively). The immunoenzymatic enzyme-linked immunosorbent assay (ELISA) methods were used to determine the insulin concentration (Insulin ELISA, DRG Instruments GmbH, Germany, sensitivity 1.76 µIU/ml) and the parathormone (PTH) concentration (Parathyroid Intact ELISA, DRG Instruments GmbH, Germany, sensitivity 1.57 pg/ml) and the enzymeimmunoassay (EIA) method was used to determine the sclerostin concentration (Human Sclerostin HS EIA Kit, TECOmedical Groupe, QUIDEL, USA, sensitivity 0.008 ng/ml). The homeostatic model assessment of insulin resistance index (HOMA-IR,) was calculated using the formula developed by Matthews et al. (1985):

\begin{eqnarray*}\text{HOMA-IR}=(\text{Insulin}[\mu \text{IU}/\text{ml}]\times \text{Glucose [mmol/l]})/22.5 \end{eqnarray*}

### Statistical methods

The data are presented as mean, standard deviation (SD), median and interquartile range. The normality of distributions was verified using the Shapiro–Wilk test. The *t*-test and Mann–Whitney *U* test were employed for normally and non-normally distributed variables, respectively, to evaluate the significance of differences between the groups. The *t*-test and Wilcoxon test were used for normally and non-normally distributed variables, respectively, to evaluate the significance of differences over time (between both terms of the measurements). It was assumed that the analysis of repeated-measures ANOVA 2x2 (time x group) will be performed if the differences in variables over time and between groups are significant. The Pearson analysis for normally distributed variables and Spearman’s rank analysis for non-normally distributed variables were used to calculate correlation coefficients.

Statistical significance was set at an alpha of 0.05 for all statistical procedures. The results were analysed statistically using the Dell Inc. (2016) Dell Statistica (data analysis software system), version 13. software.dell.com.

## Results

Table 1 presents descriptive statistics of somatic features and biochemical indices of the women examined on two terms of the study.

**Table 1:** Somatic characteristics and biochemical indices in the two study terms for the entire group of women involved in the study (*n* = 16).

Parameters	Assessment at baseline (term I)	Assessment at the end (term II)
Body mass (kg)	70.04 ± 12.48 67.55 (61.15–72.15)	69.76 ± 12.20 66.85 (61.15–72.30)
BMI (kg/m^2^)	27.79 ± 4.97 27.05 (24.85–29.05)	27.69 ± 4.98 27.15 (24.60–28.95)
Fat mass (kg)	26.26 ± 7.46 24.90 (21.70–28.70)	26.22 ± 7.66 24.90 (21.45–28.35)
Fat mass (%)	36.96 ± 3.88 36.60 (34.75–39.20)	37.02 ± 4.23 35.95 (33.95–39.60)
25(OH)D (ng/ml)	30.58 ± 9.37 29.45 (25.90–34.15)	24.73 ± 9.35 21.85 (18.65–29.15)^**^
Glucose (mmol/l)	5.42 ± 0.52 5.31 (5.14–5.63)	5.25 ± 0.52 5.28(4.90–5.42)
Insulin (µIU/ml)	12.73 ± 4.56 11.45 (9.57–14.99)	10.51 ± 2.49 10.52 (8.53–11.70)^*^
HOMA-IR	3.09 ± 1.25 3.04(2.33–3.64)	2.46 ± 0.63 2.56 (1.84–2.77)^*^
TC (mg/dl)	252.06 ± 44.11 246.50 (217.00–293.50)	255.25 ± 44.21 256.00 (232.50–283.00)
TG (mg/dl)	107.44 ± 51.80 91.00 (70.50–125.00)	97.31 ± 40.61 92.00 (66.00–114.50)
HDL-C (mg/dl)	66.10 ± 12.53 68.00 (55.50–73.45)	65.92 ± 12.66 65.85 (57.45–71.05)
LDL-C (mg/dl)	161.74 ± 39.42 155.95 (146.80–184.65)	165.61 ± 42.33 162.65 (138.55–188.95)
PTH (pg/ml)	48.53 ± 12.74 46.41 (38.44–54.76)	46.95 ± 14.72 47.11(32.92–57.31)
Sclerostin (ng/ml)	0.51 ± 0.12 0.49 (0.46–0.60)	0.57 ± 0.27 0.54 (0.36–0.69)

**Notes.**

Results are expressed as mean (SD); median (interquartile range).

* Significant difference (*p* < 0.05).

** Significant difference (*p* < 0.01).

BMI body mass index 25(OH)D 25-hydroxyvitamin D HOMA-IR homeostatic model assessment of insulin resistance index TC total cholesterol TG triglycerides HDL-C high density lipoprotein cholesterol LDL-C low density lipoprotein cholesterol PTH parathormone

Comparative analysis of these variables between the study terms revealed significant decrease in concentrations of 25(OH)D (*p* ≤ 0.0001), insulin (*p* = 0.0243), HOMA-IR (*p* = 0.0284) and the tendency to decrease in BMI (*p* = 0.0995) and TG concentration (*p* = 0.0995). No significant changes were noted for somatic features and other biochemical indices.

The women were divided into groups according to their BMI: group A (*n* = 10) subjects with normal BMI and group B (*n* = 6) subjects with BMI above normal. Table 2 presents descriptive statistics of somatic features and biochemical indices of the both groups of women and comparative analysis between these groups in two terms of the study and between the study terms.

**Table 2:** Somatic characteristics and biochemical indices in the two study terms for the group A (*n* = 10) and group B (*n* = 6) of women.

Parameters	Groups	Assessment at baseline (term I)	Assessment at the end (term II)
Body mass (kg)	A	63.25 ± 4.81^##^	63.11 ± 5.08^##^
	B	81.35 ± 13.43	80.83 ± 12.83
BMI (kg/m^2^)	A	25.25 ± 1.75^##^	25.19 ± 1.89^##^
	B	32.02 ± 5.86	31.79 ± 5.36
Fat mass (kg)	A	22.22 ± 3.19^##^	22.05 ± 2.90^##^
	B	32.98 ± 7.85	33.18 ± 8.25
Fat mass (%)	A	35.01 ± 2.91^##^	34.83 ± 2.54^##^
	B	40.20 ± 3.12	40.68 ± 4.04
25(OH)D (ng/ml)	A	32.04 ± 10.24	26.69 ± 10.63^**^
	B	28.15 ± 7.96	21.47 ± 6.19^**^
Glucose (mmol/l)	A	5.2 ± 0.31^#^	5.06 ± 0.42^#^
	B	5.78 ± 0.63	5.57 ± 0.53
Insulin (µIU/ml)	A	11.38 ± 3.97	10.09 ± 2.34
	B	14.98 ± 4.95	11.21 ± 2.79
HOMA-IR	A	2.64 ± 0.95	2.28 ± 0.59
	B	3.85 ± 1.42	2.76 ± 0.62
TC (mg/dl)	A	262.60 ± 43.41	262.60 ± 35.56
	B	234.50 ± 43.04	243.00 ± 57.45
TG (mg/dl)	A	107.00 ± 52.63	97.8 ± 37.01
	B	108.17 ± 55.35	96.50 ± 49.81
HDL-C (mg/dl)	A	66.91 ± 13.86	67.02 ± 12.55
	B	64.77 ± 11.04	64.08 ± 13.82
LDL-C (mg/dl)	A	170.55 ± 32.58	169.64 ± 34.28
	B	147.07 ± 48.35	158.90 ± 56.33
PTH (pg/ml)	A	46.22 ± 9.66	44.58 ± 13.46
	B	52.40 ± 17.05	50.89 ± 17.15
Sclerostin (ng/ml)	A	0.52 ± 0.12	0.52 ± 0.22
	B	0.51 ± 0.12	0.65 ± 0.35

**Notes.**

Results are expressed as mean ± SD.

# Significant difference between groups A and B (*p* < 0.05).

## Significant difference between groups A and B (*p* ≤ 0.01).

* Significant difference (*p* < 0.05).

** Significant difference (*p* < 0.01).

BMI body mass index 25(OH)D 25-hydroxyvitamin D HOMA-IR homeostatic model assessment of insulin resistance index TC total cholesterol TG triglycerides HDL-C high density lipoprotein cholesterol LDL-C low density lipoprotein cholesterol PTH parathormone

A comparative analysis of somatic features and biochemical indices between these groups in the first study term showed significantly lower values of body mass (*p* = 0.0201), BMI (*p* = 0.0014), fat mass (FM%) (*p* = 0.0046) and glucose (*p* = 0.0255) in group A compared to group B. Tendency to the significance of differences were recorded for HOMA-IR (*p* = 0.0596). Comparative analysis of somatic features and biochemical indices between these groups in the second term of the study revealed significantly lower values of body mass (*p* = 0.0180), BMI (*p* = 0.0020), FM (kg) (*p* = 0.0200), FM (%) (*p* = 0.0029), glucose (*p* = 0.0393) in group A compared to group B. There were no significant differences between groups A and B with respect to other somatic features and biochemical indices.

**Figure 1:**
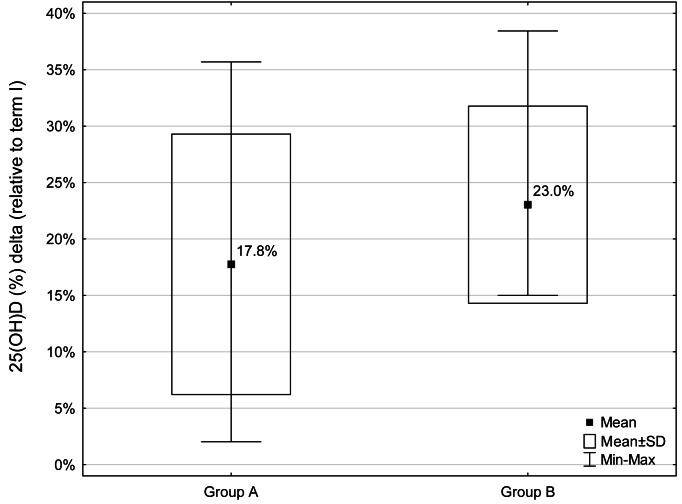
Percentage decrease (Δ) in 25(OH)D concentrations during the study period (relative to term I) for groups A and B (A - group with a normal BMI, B - group with a BMI above the norm). Decrease in 25(OH)D concentrations was observed in all participants.

Comparative analysis of somatic features and biochemical indices between the study terms in women from group A and group B showed a significant decrease only in relation to 25(OH)D concentration (*p* = 0.0009 and *p* = 0.0039, respectively). The 25(OH)D concentration decreased about 17.8% and 23% in the group A and B, respectively (Fig. 1).

Comparative analysis of the values of changes (Δ) in somatic features and biochemical indices during the study period (between terms I and II) between the two groups of women (A and B) showed no significant differences.

With respect to the variables that were measured, there was no simultaneous influence of time and group, therefore ANOVA was not carried out.

**Figure 2:**
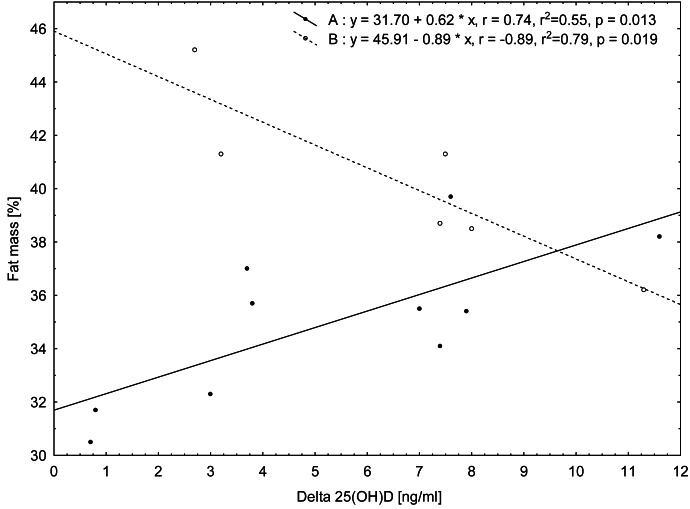
Relationship between values of change (Δ) in 25(OH)D concentrations from term I to term II and fat mass (%) measured in term I for the two groups of women according to their BMI category: group A (normal BMI) and group B (BMI above the norm).

The relationship analysis conducted for the whole group of the women showed no significant correlation between the magnitude of the reduction in 25(OH)D concentrations between the first and second terms of the study (Δ) and the somatic features and biochemical indices assessed at the beginning of the study (term I). Only a tendency for positive correlations between changes (Δ) 25(OH)D and insulin concentration on the first study term (*r* = 0.44, *p* = 0.0888) was noted. However, in the division into groups (A and B), in group A significant positive relationship of Δ25(OH)D concentration (reduction between terms I and II) with body mass (*r* = 0.70, *p* = 0.0249) measured on the first study term was found. In the case of FM, measured on the first study term, the relationships of this variable with Δ25(OH)D concentration (reduction between terms I and II) were found to be positive in group A [FM(%) *r* = 0.74, *p* = 0.0135; FM (kg) *r* = 0.79, *p* = 0.0062] and negative in group B [FM(%) *r* =  − 0.89, *p* = 0.0187; FM(kg) *r* =  − 0.84, *p* = 0.0342, Fig. 2].

On the basis of the correlation results, it can be stated that in group A the decrease in 25(OH)D concentration during the study period is greater when the fat mass content is higher, whereas in group B, the higher the fat mass, the smaller the decrease in 25(OH)D concentration.

The analysis of the relationships for the whole group of women (*n* = 16) showed significant negative correlations of Δ (difference between the first and second terms of the study) glucose and tendention to correlation of Δinsulin, ΔHOMA-IR with sclerostin concentrations measured at the term I (*r* =  − 0.50, *p* = 0.0490; *r* =  − 0.48, *p* = 0.0621; *r* =  − 0.45, *p* = 0.0825, respectively) and tendency to correlation of ΔPTH concentrations with FM (%) value measured at the term I (*r* =  − 0.46, *p* = 0.0730).

The analysis of correlations of changes during the study period of biochemical indicators and somatic features, carried out for the whole group of women (*n* = 16), showed significant positive relationships of ΔTC with: ΔTG (*r* = 0.51, *p* = 0.0449), ΔHDL-C (*r* = 0.65, *p* = 0.0069), ΔLDL-C (*r* = 0.94, *p* ≤ 0.0001), Δinsulin (*r* = 0.53, *p* = 0.0338), then ΔLDL-C with: Δinsulin (*r* = 0.59, *p* = 0.0159) and ΔHOMA-IR (*r* = 0.56, *p* = 0.0244), as well as Δglucose with Δsclerostin (*r* = 0.64, *p* = 0.0076). Moreover, a tendency of relationship of ΔFM (%) with: Δinsulin (*r* = 0.48, *p* = 0.0588), ΔHOMA-IR (*r* = 0.52, *p* = 0.0394) and then Δ25(OH)D with Δinsulin (*r* = 0.43, *p* = 0.0969) was found.

## Discussion

In our study of postmenopausal women, we recorded a significant decrease in 25(OH)D concentration in the period from autumn to winter (the mean value decreased by about 19.7% and the median by 20.8%). Taking into account the fact that vitamin D contributes to the metabolism of carbohydrates and lipids, we expected that during the study period we would also observe a deterioration in these processes. Paradoxically, we also observed a significant decrease in the insulin resistance index.

With regard to seasonal changes in 25(OH)D concentration, it is difficult to compare the results of our research to those obtained by other authors, because these changes in 25(OH)D concentration in blood can be influenced by numerous factors, such as climate zone and related UV intensity. Andersen et al. (2013) also conducted a study on 25(OH)D concentrations in winter and late summer (February–March and August–September), and also in February–March the following year for Danish women (aged 70–75 years). Their findings show that between summer and winter, the level of vitamin D (median) decreased on average by approximately 25%. These results are comparable to the changes recorded in our study. The authors concluded that in order to reach suboptimal level of 25(OH)D in blood serum during winter (in line with the recommended level of approximately 50 nmol/l, 20 ng/dl), it is necessary to reach a level of around 100 nmol/l (40 ng/dl) during summer. In our study, we observed that at the beginning of the winter, the average 25(OH)D was higher than 20 ng/dl if the women’s 25(OH)D was higher than 25 ng/dl in early autumn. However, it should be noted that in our study we measured 25(OH)D at the beginning of the winter season (December), while in the study by Andersen et al. (2013) it was measured during the February-to-March period. Similarly, in studies conducted in an Estonian population (women and men aged 25-70) it was observed that the average concentration of 25(OH)D in women in the winter period (January–March) was 26.3% lower than in the summer (September) (Kull Jr et al., 2009). In these studies, it was observed that the 25(OH)D concentrations were strongly correlated with sunbathing habits, both in winter and in summer time. Andersen et al. (2013) observed that vitamin D intake from supplements (*p* < 0.0001) and diet (*p* = 0.002) were also determinants of the change in vitamin D status in the period from summer to winter.

The main source of vitamin D in the human body in winter is food, especially fish. Nakamura et al. (2000) study conducted in a Japanese female population revealed that fish consumption was significantly associated with 25(OH)D serum concentrations (*p* < 0.001) and the frequency of fish consumption (≥ 4 times/wk) may be an important determinant of 25(OH)D level in the winter. In the women in our study, fish consumption was relatively low. The majority of the women declared that they consume fish once a week (*n* = 13) or less (*n* = 2) and only one woman declared fish consumption twice a week.

The main finding of this study was to reveal a significant relationship between seasonal changes in 25(OH)D concentrations and body fat percentage measured at the beginning of the study (the first term of the study—the autumn period) in both groups of postmenopausal women. However, on the basis of these results it can be concluded that there is a different direction of these correlations, depending on BMI. In women with normal BMI (group A), the decrease in 25(OH)D concentration was greater when the body fat percentage was higher, whereas in women with a BMI value above the reference values (group B), the higher the fat percentage, the smaller the decrease in 25(OH)D concentration. This finding may suggests that in women with higher BMI and a higher percentage of fat mass, fat tissue can be a reservoir of vitamin D that attenuates its decrease, however, it needs further investigations.

The relationships of a low serum 25(OH)D concentration with an increased fat mass (Tosunbayraktar et al., 2005), and with total vitamin D in omental and subcutaneous fat in both obese individuals and those with normal body mass (Carrelli et al., 2017) were confirmed by several studies. Carrelli et al. (2017), who used fat tissue biopsy, advanced the hypothesis that a large amount of this tissue in obese women (18–70 years old) is a reservoir of vitamin D. Vitamin D content in adipose tissue may therefore predispose obese individuals to a reduced serum 25(OH)D level. In our study, although it was confirmed that there is the significant relationship between seasonal variations (Δ) in 25(OH)D and FM(%) in the autumn period in both groups of women, it is interesting that there are different directions of these correlations. We did not observe any significant relationship of 25(OH)D concentration with the body fat percentage in the whole group of women in both study terms. However, it should be emphasized that other factors, such as the frequency of women’s outdoor activities, especially during the first study period, may have also influenced 25(OH)D concentrations.

In our study, we did not observe any associations of 25(OH)D with lipid metabolism indices and any significant changes in lipid profile from the beginning of autumn to winter. The associations of 25(OH)D with TC, HDL-C, LDL-C (Jungert, Roth & Neuhäuser-Berthold, 2015) and with TG‘s (Grimnes et al., 2011) have been confirmed in prevous studies. However, Jungert, Roth & Neuhäuser-Berthold (2015) observed these relationships only at 25(OH)D levels ≥62 nmol/l, independent of body composition and lifestyle. Moreover, Grimnes et al. (2011) found that additional supplementation with vitamin D had no effects on TG and other lipids concentrations.

We did confirm a significant decrease in insulin and HOMA-IR during the study period, which indicates an improvement in insulin sensitivity. Seasonal changes in insulin sensitivity and lipid profile were observed in studies of other authors investigating elderly people (Berglund et al., 2012; Skutecki et al., 2019). In these studies, authors have explained their results by the variability of the external temperature and nutritional habits leading to changes in body composition. In our study, we wanted to check whether the seasonal changes in 25(OH)D concentration would be related to changes in insulin sensitivity and lipid profile. Due to the seasonal decrease in 25(OH)D and its contribution to carbohydrate and lipid metabolism, we expected changes in the opposite direction. Vitamin D modulates glycemic homeostasis by modulating the secretion of insulin by *β*-cells and increasing glucose uptake in tissues (Greco, Lenzi & Migliaccio, 2019; Girgis et al., 2013). It was demonstrated that 1,25(OH)_2_D_3_ is a significant contributor to the expression of insulin receptors, insulin signaling in skeletal muscles and the reduction in hyperglycemia and hyperinsulinemia (Girgis et al., 2013). However, in our study we did not identify significant correlations between 25(OH)D concentrations and carbohydrate metabolism indices; we only noted a tendency towards a positive correlation of 25(OH)D changes (Δ) with insulin concentration measured on the first study term (*p* = 0.0888). This may indicate a greater decrease in 25(OH)D concentration in subjects with lower insulin sensitivity. We have also noticed a trend of a positive relationship between the decrease (Δ) in 25(OH)D concentration and the decrease (Δ) in insulin concentration (*p* = 0.0969) over the study period.

The explanation that improved insulin sensitivity (reduction in insulin and HOMA-IR levels) in our study in the period from early autumn to winter (contrary to our expectations and despite the reduction in 25(OH)D levels) can hypothetically be based on the composition of several factors—drop in the external temperature and an increased basic metabolism leading to body mass reduction or religious-based limitations in food intake because of completing the study in the pre-Christmas period (Skutecki et al., 2019).

In our study, we did not observe any significant changes in the PTH concentrations, neither in the women as a whole nor when divided by groups. PTH is the main stimulator of vitamin D hydroxylation in the kidneys, while vitamin D exerts a negative influence on PTH secretion. A low level of 25(OH)D leads to reduced intestinal calcium absorption efficiency and the body reacts by increasing the secretion of PTH (Heaney, 2008). However, there are suggestions that the threshold of serum 25(OH)D, where serum PTH starts to rise, is about 75 nmol/l (30 ng/ml), according to most surveys (Lips, 2006).

In our study, we evaluated the concentration of sclerostin. However, changes in 25(OH)D concentrations between the terms of the study in postmenopausal women were not significantly related to the change in serum sclerostin or its concentrations at the beginning of the study. Azzam et al. (2019) in the cross-sectional study of subjects with obesity and normal body mass hypothesized that the relationship between sclerostin and vitamin D levels has an important role in the link between obesity and bone metabolism. Sclerostin is a glycoprotein produced by osteocytes and to a lesser extent by other cell types (the kidneys, vessels). By inhibiting the canonical signal pathway of the Wnt/*β*-catenin tract, it is a negative regulator of osteoblast activity (Clarke & Drake, 2013). Apart from its main activity related to bone resorption, sclerostin is also involved in carbohydrate metabolism (Daniele et al., 2015). Such a relationship may be indicated by the significant negative correlations we observed between the concentrations of sclerostin in term I and the change in the concentrations of glucose and tendency to relationship with insulin and HOMA-IR and a high positive correlation between the change in the sclerostin concentration and the change in glucose concentration between the study terms. Daniele et al. (2015) demonstrated that sclerostin levels are increased in pre-diabetic patients and correlated with insulin levels and insulin resistance measured using the oral glucose tolerance test (OGGT) and with the euglycemic-hyperinsulinemic clamp. The authors concluded that in the initial stage of glucose intolerance and insulin resistance, sclerostin may play a role in the action of insulin and its clearance and put forward the hypothesis that it may be involved in the Wnt signaling of liver glucose metabolism. In a cohort study, Yu et al. (2017) also observed a relationship between sclerostin concentrations and fasting insulin levels and HOMA-IR, but found no clear link to the risk of type 2 diabetes.

The limitation of the study is that we did not study women’s diet in the two terms - at the beginning and at the end of the study. This would have allowed us to assess the influence of diet on the indices being studied (especially insulin concentration). Moreover, the study was carried out on a small sample. On the other hand, the strength of this study was that it excluded participants with factors such as drugs or supplements which would have impacted the data, and therefore our study limited these counfounding factors. It is also worth noting that the women participating in the study declared that they did not engage in systematic intensive physical activity, which may be confirmed by the absence of changes in sclerostin concentrations (Janik et al., 2018).

## Conclusion

The results of this study indicate a significant seasonal decrease in 25(OH)D in the autumn-winter period, which in women with normal BMI was greater as the body fat percentage became higher, while in the higher BMI group, a higher body fat percentage tracked with a smaller decrease in 25(OH)D. Changes in 25(OH)D concentrations did not significantly affect the concentration of carbohydrate and lipid metabolism indices. Therefore, it may be environmental or lifestyle-related factors, such as nutritional behaviour, that may have had a greater impact on these indices.

##  Supplemental Information

10.7717/peerj.11341/supp-1 Supplemental Information 1 Raw data for serum concentrations of 25(OH)D, sclerostin, carbohydrate and lipid metabolic indices in postmenopausal women in two terms (autumn - winter)

## Additional Information and Declarations

### Data Availability

The following information was supplied regarding data availability: The raw measurements are available in the Supplementary File.
